# Neuroprotective effect of angiotensin II receptor blockers on the risk of incident Alzheimer’s disease: A nationwide population-based cohort study

**DOI:** 10.3389/fnagi.2023.1137197

**Published:** 2023-03-06

**Authors:** Hyun Woo Lee, Seungyeon Kim, Youngkwon Jo, Youjin Kim, Byoung Seok Ye, Yun Mi Yu

**Affiliations:** ^1^Department of Pharmaceutical Medicine and Regulatory Sciences, Colleges of Medicine and Pharmacy, Yonsei University, Incheon, Republic of Korea; ^2^Department of Pharmacy and Yonsei Institute of Pharmaceutical Sciences, College of Pharmacy, Yonsei University, Incheon, Republic of Korea; ^3^College of Pharmacy, Dankook University, Cheonan, Republic of Korea; ^4^Department of Neurology, College of Medicine, Yonsei University, Seoul, Republic of Korea

**Keywords:** Alzheimer’s disease, renin-angiotensin system, angiotensin II receptor blocker, blood–brain barrier, neuroprotective agents

## Abstract

**Background:**

Recent studies on renin-angiotensin system (RAS) inhibitors have reported a reduced risk of Alzheimer’s disease (AD). Nevertheless, the effect of RAS inhibitor type and blood–brain barrier (BBB) permeability on the risk of AD is still unknown.

**Objectives:**

To assess the effects of RAS inhibitors on the risk of AD based on the type and BBB permeability and investigate the cumulative duration-response relationship.

**Methods:**

This was a population-based retrospective cohort study using the Korean Health Insurance Review and Assessment database records from 2008 to 2019. The data of patients diagnosed with ischemic heart disease between January 2009 and June 2009 were identified for inclusion in the analyses. Propensity score matching was used to balance RAS inhibitor users with non-users. The association between the use of RAS inhibitors and incident AD was evaluated using a multivariate Cox proportional hazard regression model. The results are presented in adjusted hazard ratios (aHRs) and 95% confidence intervals (CIs).

**Results:**

Among the 57,420 matched individuals, 7,303 developed AD within the follow-up period. While the use of angiotensin-converting enzyme inhibitors (ACEIs) was not significantly associated with AD risk, the use of angiotensin II receptor blockers (ARBs) showed a significant association with reduced risk of incident AD (aHR = 0.94; 95% CI = 0.90–0.99). Furthermore, the use of BBB-crossing ARBs was associated with a lower risk of AD (aHR = 0.83; 95% CI = 0.78–0.88) with a cumulative duration-response relationship. A higher cumulative dose or duration of BBB-crossing ARBs was associated with a gradual decrease in AD risk (*P for trend < 0.001*). No significant association between the use of ACEIs and the risk of AD was observed regardless of BBB permeability.

**Conclusion:**

Long-term use of BBB-crossing ARBs significantly reduced the risk of AD development. The finding may provide valuable insight into disease-modifying drug options for preventing AD in patients with cardiovascular diseases.

## Introduction

During the past few decades, Alzheimer’s disease (AD) has emerged as a leading global health concern (Alzheimer’s Disease International, 2019). Despite constant efforts to develop new drugs, the complexity of pathologies has left no promising treatments for AD (Cummings et al., 2014; Bachurin et al., 2017). Owing to the limitations and uncertainty of current treatments, targeting modifiable risk factors for preventing AD incidence and delaying progression has gained importance in recent years. Management of hypertension with antihypertensive drugs has been recommended for the primary prevention of AD (Crous-Bou et al., 2017; Livingston et al., 2020; Hefner et al., 2021). The experimental results, however, remain controversial, and further analyzes are required to reach a general consensus on the use of antihypertensive drugs (Yasar et al., 2013; Chuang et al., 2014; Walker et al., 2020). Among the antihypertensive agent classes, renin-angiotensin system (RAS) inhibitors, particularly angiotensin-converting enzyme inhibitors (ACEIs) and angiotensin II type 1 receptor blockers (commonly used as angiotensin II receptor blockers, ARBs), have been reported to have potential benefits on reducing the risk of incident AD (Li et al., 2010; Davies et al., 2011; Barthold et al., 2018).

RAS is a hormonal system responsible for regulating blood pressure (BP), fluid balance, electrolyte homeostasis, and vascular resistance. This system is mediated by angiotensin (Ang) ligands that interact with various receptors, including angiotensin II type 1 receptor (AT1R), angiotensin II type 2 receptor (AT2R), angiotensin IV receptor (AT4R), and Mas receptor (MASR; Gebre et al., 2018). In addition to the peripheral RAS, the receptors in the central nervous system (CNS) are involved in oxidative stress, neuroinflammation, and neuronal apoptosis, causing neurodegeneration (Abiodun and Ola, 2020; Xu et al., 2021). Accordingly, blood–brain barrier (BBB)-crossing RAS inhibitors that block neurodegenerative pathways may provide neuroprotective effects in brain disorders including AD.

A few clinical studies have reported that CNS penetration of RAS inhibitors was associated with reduced cognitive decline and a lower conversion rate from mild cognitive impairment (MCI) to AD (Ohrui et al., 2004; Sink et al., 2009; O’Caoimh et al., 2014; Wharton et al., 2015). However, other studies have shown unclear relationship between BBB permeability and increased neuroprotection by RAS inhibitors (Hebert et al., 2013; Fazal et al., 2017). Considering that most studies focused on ACEIs and the results were inconclusive, further studies are essential in clarifying the effect of BBB-crossing RAS inhibitors, independent from blood pressure lowering effect, on the risk of AD incidence.

This study aimed to assess the effects of RAS inhibitors on the risk of incident AD by using a longitudinal national health insurance database in Korea. In addition to comparing the effect of RAS inhibitors on AD based on class and BBB permeability, we further investigated the cumulative duration-response relationship.

## Materials and methods

### Study design and data source

This population-based retrospective cohort study was conducted using Health Insurance Review and Assessment Service (HIRA) data collected for reimbursing healthcare providers between 2008 and 2019. In 2000, South Korea implemented a centralized single-insurer system, achieving healthcare coverage for almost the entire Korean population (Kwon, 2009). Universal coverage of health insurance allowed HIRA to develop a database containing socio-economic information and clinical details, including healthcare services, diagnoses, and prescriptions of 50 million beneficiaries (Kim et al., 2017). Restricted access to encrypted datasets is granted to generate public statistics and clinical research.

This study was approved by the Institutional Review Board (IRB) of Yonsei University (IRB number: 7001988-202,004-HR-846-01E). The requirement for informed consent was waived owing to the anonymity of the data and the retrospective design of the study.

### Study population

The study population consisted of Korean National Health Insurance beneficiaries aged 60 years or older who were diagnosed with ischemic heart disease (IHD) during the identification period (January 1, 2009, to June 30, 2009). The diagnosis of IHD was defined as the use of diagnostic codes for angina or myocardial infarction (MI) according to the International Classification of Diseases 10th Revision (ICD-10): I20.0-I22.0. Study participants were followed from the index date (July 1, 2009) to the occurrence of the outcome, the date of death, or the end of the claims record (December 31, 2019), whichever occurred first.

Users of RAS inhibitors were defined as patients with the first prescription record of RAS inhibitors during the identification period. Patients who had never been prescribed RAS inhibitors during the study period were classified as non-users. Patients were excluded from the study based on the following criteria: (1) RAS inhibitor use prior to the identification period; (2) first use of RAS inhibitors after the identification period; (3) death record or last claims record prior to follow-up; and (4) diagnosis of dementia (F00-F03, G30-G31), MCI (F06.7, R41), or Parkinson’s disease (G20) before the follow-up. The outcome variable was assessed with a 1 year lag time to control and minimize reverse causality (Rea et al., 2005).

### Exposure assessment

The RAS inhibitors included in this study were ACEIs and ARBs, based on the Anatomic Therapeutic Chemical (ATC) classification system provided by the World Health Organization (WHO) Collaborating Center for Drug Statistics Methodology (WHO Collaborating Centre for Drug Statistics Methodology, 2021). RAS inhibitor users were further categorized into the following four subgroups according to the type of RAS inhibitors and BBB permeability: poor BBB-crossing ACEIs (alacepril, benazepril, cilazapril, enalapril, imidapril, moexipril, and quinapril), BBB-crossing ACEIs (captopril, delapril, fosinopril, lisinopril, perindopril, ramipril, temocapril, trandolapril, and zofenopril), poor BBB-crossing ARBs (eprosartan, irbesartan, losartan, and olmesartan), and BBB-crossing ARBs (azilsartan, candesartan, fimasartan, telmisartan, and valsartan). Categorization was based on available evidence from previous analysis and review research. Drugs were considered poor BBB-crossing if the BBB permeability were inconclusive and/or if they showed relatively low lipophilicity (Oka et al., 1988; Takai et al., 2004; Sink et al., 2009; Wharton et al., 2012; Michel et al., 2013; Yagi et al., 2013; Kim et al., 2015; Alzahrani et al., 2020; Ho et al., 2021; Ouk et al., 2021; Jo et al., 2022).

The dosage, frequency, and prescription days of RAS inhibitors during the identification and follow-up periods were multiplied and used as the cumulative dose. The cumulative doses were then converted into the cumulative defined daily dose (DDD) using the ATC-DDD toolkit provided by the WHO (WHO Collaborating Centre for Drug Statistics Methodology, 2021), as the claims database does not provide information regarding individual body weight. DDD is the assumed average maintenance dose per day for a drug used for its main indication in adults. The cumulative exposure duration and daily equivalent dose of RAS inhibitors were defined as the sum of the prescription days and ratio of cumulative DDD to cumulative exposure duration, respectively.

### Definition of outcome

The primary outcome was the incidence of AD during the follow-up period. To enhance the accuracy of AD outcome measurement, new onset AD was defined as the presence of two or more prescription records of any AD treatment drug with an AD diagnostic code (ICD-10 F00, G30) generated from neurology or psychiatry department. Diagnostic codes obtained without the restriction on departments were included in the sensitivity analysis. The AD treatment drugs included donepezil, galantamine, rivastigmine, and memantine, which have been approved by the Food and Drug Administration for AD. Aducanumab was excluded because it was newly approved in June 2021. The outcome date was defined as the first occurrence of an AD diagnostic code within the follow-up period.

### Covariates

Sociodemographic data, including patients’ age, sex, and type of insurance (health insurance and medical aid), were collected during the identification period. Comorbid diseases and concomitant medications were recorded up to the date of outcome, death, or last claims record. Patients were defined as having comorbid diseases or concurrent medications when diagnostic or drug codes appeared annually during the follow-up period.

In this study, comorbid diseases reported to be potential risk factors of AD included atrial fibrillation (AF), atherosclerosis, bipolar disorder, cerebrovascular disease (hemorrhagic infarction, ischemic cortical infarction, and vasculopathy), depression, diabetes mellitus (DM), dyslipidemia, hypertension, Parkinson’s disease (PD), schizophrenia, sleep disorder, traumatic brain injury (TBI), and vascular dementia (VD; Profenno et al., 2010; Ballard et al., 2011; Xu et al., 2015). A list of ICD-10 codes for comorbid diseases is presented in Supplementary Table S1. Concomitant medications reported as potential protective or risk factors of AD included antidepressants, antiepileptics, antihistamines, antiparkinsonian agents, antipsychotics, antispasmodics, beta-blockers, benzodiazepines, bladder antimuscarinics, dihydropyridine calcium channel blockers (CCB-D), non-dihydropyridine calcium channel blockers (CCB-ND), 3-hydroxy-3-methylglutaryl coenzyme A (HMG-CoA) reductase inhibitors, skeletal muscle relaxants, and zolpidem (Carnahan et al., 2006; Risacher et al., 2016; American Geriatrics Society Beers Criteria® Update Expert Panel, 2019). Diseases or medications with a frequency of less than 30 in the population were excluded from the covariates (Yu et al., 2017). A detailed list of the concomitant medications is provided in Supplementary Table S2.

### Statistical analyzes

We adopted propensity score matching (PSM) to control covariate imbalance and minimize treatment assignment bias with a multivariate logistic regression model. RAS inhibitor users were matched to non-users in a 1:1 ratio with no replacement, using the greedy matching method. The caliper width was set to 0.2 of the pooled standard deviation of the logit of the propensity score (Austin, 2011). The matching variables included age, sex, type of insurance, follow-up duration, comorbid diseases, and concurrent medications. PSM was validated by performing balance diagnostics using standardized mean difference (SMD). The absolute value of the SMD less than 0.1 was considered well balanced. Graphical distributions of propensity scores in cohorts before and after matching are presented in Supplementary Figure S1.

Demographics and clinical characteristics of RAS inhibitor users and non-users were compared using descriptive statistics. Categorical variables were presented as frequencies with percentages using Pearson’s chi-squared test. Continuous variables were described as mean ± standard deviation (SD) using the Student’s *t*-test. The incidence of AD per 1,000 person-years was calculated by dividing the number of incident AD cases by the total follow-up person-years and multiplying the rate by 1,000.

The proportional hazard assumption for the BBB-crossing ARBs was graphically validated using the log minus log plot of the Kaplan–Meier estimation (Supplementary Figure S2). The hazard ratio (HR) and 95% confidence interval (CI) of the AD incidence were estimated using multivariate Cox proportional hazard regression models adjusted for sex, age, insurance type, follow-up period, comorbid diseases, and concurrent medications. The adjusted hazard ratio (aHR) between RAS inhibitor use and incident AD was computed based on RAS type and BBB permeability. Further analyzes of cumulative dose and duration-dependent responses were performed according to the cumulative DDD, cumulative exposure duration, and daily equivalent dose with a trend test using the Cox model. The cumulative hazard of AD is also graphically shown using Kaplan–Meier curves. Subgroup analyzes to identify the effect of RAS inhibitors on AD based on the type and BBB permeability within the sex were conducted.

Sensitivity analyzes were conducted with four different designs for the index date, lag time, outcome definition, and exclusion criteria. First, the index date was shifted to July 1, 2010, and July 1, 2011. Second, the lag time of the outcome was extended to 3 and 5 years. Incidents during the lag time were excluded. Third, the AD incidence was re-defined with the following definitions: (1) AD diagnostic code (no restriction on departments) with at least two or more AD treatment prescriptions; and (2) AD diagnostic code with a neurology or psychiatry department subject code. Finally, the exclusion criteria were expanded with the following conditions for the entire study period 2008–2019: (1) presence of PD diagnostic code, and (2) concurrent use of ACEI and ARB. The study population was re-matched, and the outcome, comorbidity, and exposure to medications were re-assessed based on the new four designs of sensitivity analysis. Statistical analyzes were performed using the SAS software (version 9.4; SAS Institute, Cary, NC, United States). Pooled estimation with a value of *p* < 0.05 was considered significant.

## Results

### Baseline characteristics

**Figure 1:**
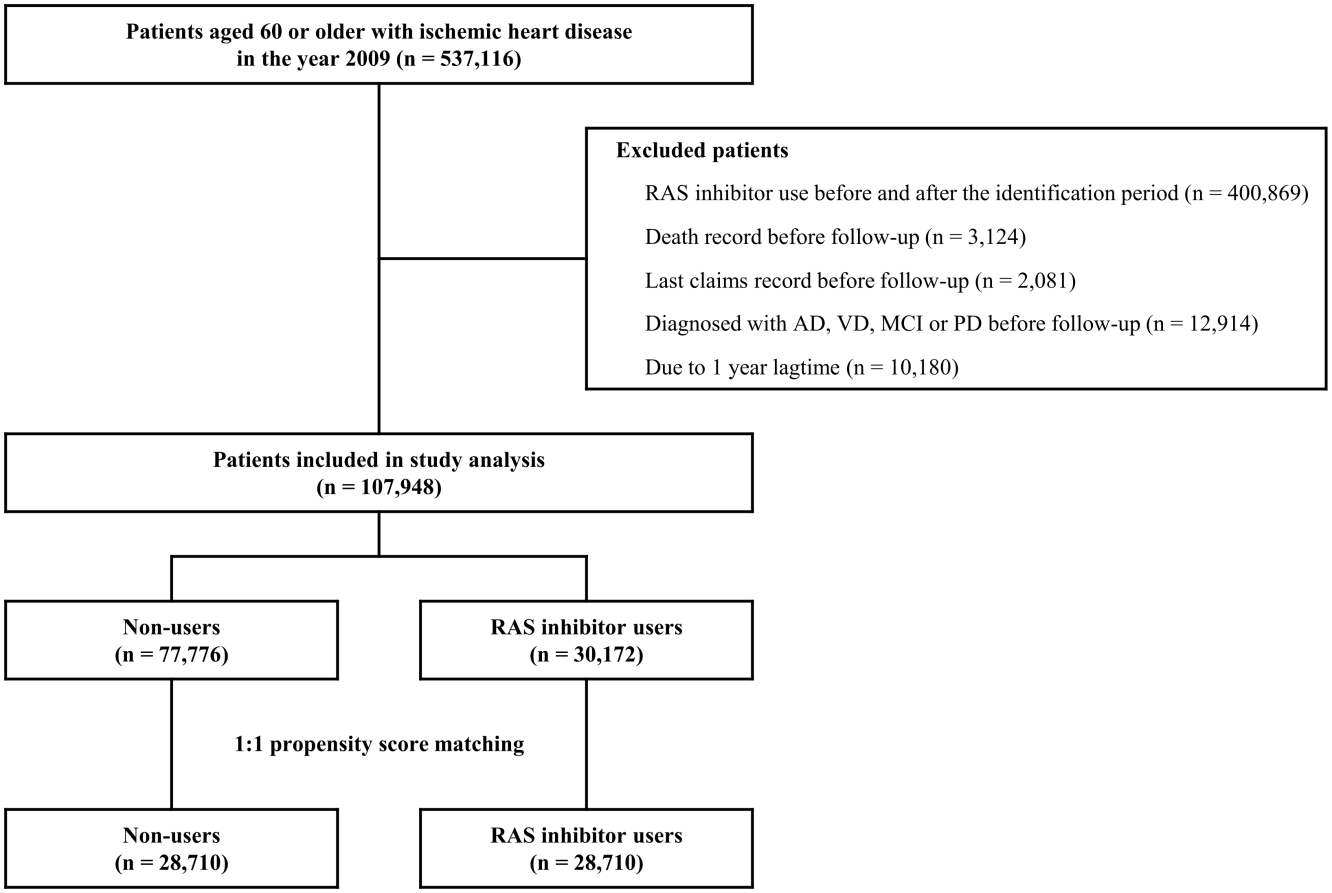
Flow chart of study population inclusion. AD, Alzheimer’s disease; MCI, mild cognitive impairment; PD, Parkinson’s disease; RAS, renin-angiotensin system; VD, vascular dementia.

The cohort of patients diagnosed with IHD between January 2009 and June 2009 consisted of 537,116 participants. After the eligibility assessment and PSM, 57,420 patients with 490,384 person-years were identified for the analysis (Figure 1). The demographic and clinical characteristics of the study population are summarized in Table 1. The mean ± SD age was 69.6 ± 6.8 years and 69.9 ± 6.8 for the matched RAS inhibitor users and non-users, respectively; the proportion of the male participants was 45.0 and 46.2% in RAS inhibitor users and non-users, respectively. The mean ± SD follow-up duration was 8.6 ± 2.9 years for RAS inhibitors users and 8.5 ± 2.9 years for non-users. Among the comorbid diseases, hypertension and dyslipidemia had a high prevalence of 61.0 and 48.0%, respectively.

**Table 1:** Baseline characteristics of the study population.

	Before PSM^a^ (*N* = 107,948)			After PSM^a^ (*N* = 57,420)		
Characteristics	Non-users, *N* (%) (*N* = 77,776)	RAS inhibitor users, *N* (%) (*N* = 30,172)	Standardized difference	Non-users, *N* (%) (*N* = 28,710)	RAS inhibitor users, *N* (%) (*N* = 28,710)	Standardized difference
Sex			0.0308			0.0241
Men	36,133 (46.5)	13,555 (44.9)		13,275 (46.2)	12,930 (45.0)	
Women	41,643 (53.5)	16,617 (55.1)		15,435 (53.8)	15,780 (55.0)	
Age, years, mean (SD)	69.1 (6.9)	69.7 (6.8)	0.0885	69.9 (6.8)	69.6 (6.8)	−0.0311
Under 65 years	22,795 (29.3)	7,606 (25.2)		7,030 (24.5)	7,376 (25.7)	
Between 65 and 80 years	48,262 (62.1)	19,784 (65.6)		18,929 (65.9)	18,678 (65.1)	
Over 80 years	6,719 (8.6)	2,782 (9.2)		2,751 (9.6)	2,656 (9.3)	
Insurance type			−0.1030			0.0035
Health insurance	72,704 (93.5)	27,367 (90.7)		26,190 (91.2)	26,218 (91.3)	
Medical aid	5,072 (6.5)	2,805 (9.3)		2,520 (8.8)	24,92 (8.7)	
Comorbidity						
Atherosclerosis	4,371 (5.6)	2,062 (6.8)	0.0503	1,903 (6.6)	1,898 (6.6)	−0.0007
Atrial fibrillation	5,452 (7.0)	3,053 (10.1)	0.1113	2,834 (9.9)	2,699 (9.4)	−0.0159
Bipolar disorder	4,146 (5.3)	1,982 (6.6)	0.0524	1,870 (6.5)	1,825 (6.4)	−0.0064
Cerebrovascular disease	13,569 (17.5)	6,379 (21.1)	0.0938	6,000 (20.9)	5,919 (20.6)	−0.0070
Depression	10,535 (13.6)	4,398 (14.6)	0.0297	4,092 (14.3)	4,155 (14.5)	0.0063
Diabetes mellitus	18,969 (24.4)	9,286 (30.8)	0.1433	8,497 (29.6)	8,435 (29.4)	−0.0047
Dyslipidemia	35,326 (45.4)	14,601 (48.4)	0.0596	13,802 (48.1)	13,731 (47.8)	−0.0050
Hypertension	32,455 (41.7)	18,933 (62.8)	0.4305	17,527 (61.1)	17,471 (60.9)	−0.0040
Parkinson’s disease	2,287 (2.9)	887 (2.9)	0.0000	828 (2.9)	847 (3.0)	0.0039
Schizophrenia	2,068 (2.7)	944 (3.1)	0.0280	867 (3.0)	885 (3.1)	0.0036
Sleep disorder	8,671 (11.2)	3,539 (11.7)	0.0182	3,336 (11.6)	3,369 (11.7)	0.0036
Traumatic brain injury	2,108 (2.7)	974 (3.2)	0.0305	889 (3.1)	902 (3.1)	0.0026
Vascular dementia	11,981 (15.4)	5,411 (17.9)	0.0679	4,981 (17.4)	5,032 (17.5)	0.0047
Concurrent medication						
Antidepressants	3,410 (4.4)	1,358 (4.5)	0.0057	1,249 (4.4)	1,284 (4.5)	0.0059
Antiepileptics	412 (0.5)	222 (0.7)	0.0260	206 (0.7)	199 (0.7)	−0.0029
Antihistamines	2,659 (3.4)	1,196 (4.0)	0.0289	1,120 (3.9)	1,131 (3.9)	0.0020
Antiparkinsonian agents	693 (0.9)	315 (1.0)	0.0156	275 (1.0)	295 (1.0)	0.0070
Antipsychotics	4,292 (5.5)	1,976 (6.6)	0.0433	1,841 (6.4)	1,850 (6.4)	0.0013
Antispasmodics	795 (1.0)	315 (1.0)	0.0022	281 (1.0)	308 (1.1)	0.0093
Benzodiazepines	7,013 (9.0)	2,240 (7.4)	−0.0580	2,163 (7.5)	2,191 (7.6)	0.0037
Beta-blockers	19,059 (24.5)	7,167 (23.8)	−0.0176	7,481 (26.1)	7,017 (24.4)	−0.0372
Bladder antimuscarinics	2,983 (3.8)	1,287 (4.3)	0.0218	1,199 (4.2)	1,223 (4.3)	0.0042
CCB (Dihydropyridine)	19,853 (25.5)	7,999 (26.5)	0.0225	7,896 (27.5)	7,917 (27.6)	0.0016
CCB (Non-dihydropyridine)	13,095 (16.8)	2,499 (8.3)	−0.2603	2,629 (9.2)	2,499 (8.7)	−0.0159
HMG-CoA reductase inhibitors	32,959 (42.4)	13,384 (44.4)	0.0400	12,624 (44.0)	12,603 (43.9)	−0.0015
Skeletal muscle relaxants	908 (1.2)	406 (1.4)	0.0160	382 (1.3)	389 (1.4)	0.0021
Zolpidem	3,085 (4.0)	1,244 (4.1)	0.0079	1,183 (4.1)	1,197 (4.2)	0.0024
Follow-up, years, mean (SD)	8.7 (2.9)	8.6 (2.9)	−0.0379	8.5 (2.9)	8.6 (2.9)	0.0109

CCB, calcium channel blocker; HMG-CoA, 3-hydroxy-3-methylglutaryl coenzyme A; *N*, number; PSM, propensity score matching; RAS, renin-angiotensin system; SD, standard deviation. ^a^Matched by sex, age, type of insurance, comorbid disease, concurrent medications, and follow-up duration presented in this table.

Before matching, significant differences in baseline characteristics were observed for the type of insurance, hypertension, DM, AF, and CCB-ND. Post-PSM results showed that the absolute SMD values for all variables were remodeled below 0.1. This indicated that the differences between the covariates were statistically well balanced.

### Renin-angiotensin system inhibitor use and AD risk

A total of 7,303 new AD cases were observed, with an overall incidence of 14.9 per 1,000 person-years. The median time of censoring was 10.5 years (interquartile range, 8.3–10.5 years) in patients with AD.

The use of RAS inhibitors was not significantly associated with AD risk. Females showed an increased risk of AD. The population aged 65 years or older had an increased risk of AD compared to those aged 60–64 years (aHR = 2.61; 95% CI = 2.41–2.83), and the risk was even greater in those aged 80 years or older (aHR = 5.71; 95% CI = 5.16–6.31). Comorbid diseases including atherosclerosis, AF, bipolar disease, cerebrovascular disease, depression, DM, dyslipidemia, hypertension, PD, schizophrenia, sleep disorder, TBI, and VD were all significant risk factors for AD incidence. Prescriptions of antidepressants, antiepileptics, antihistamines, antiparkinsonian agents, antipsychotics, antispasmodics, benzodiazepines, beta-blockers, benzodiazepines, bladder antimuscarinics, dihydropyridine CCB-D, CCB-ND, skeletal muscle relaxants, and zolpidem were also associated with an increased risk of AD. HMG-CoA reductase inhibitors were observed to be significant protective factors against the incidence of AD (Table 2).

**Table 2:** Cox regression analysis of the association between incident Alzheimer’s disease and renin-angiotensin system inhibitors and confounding factors (*N* = 57,420).

Characteristics	Number of subjects	Person-years	Number of events	Incidence rate^a^	Unadjusted HR (95% CI)	Adjusted HR (95% CI)^b^	value of *p*
RAS inhibitors
Non-users	28,710	244,738	3,689	15.07	Ref.	Ref.	
Users	28,710	245,646	3,614	14.71	0.97 (0.93–1.02)	0.99 (0.94–1.03)	0.5886
Sex
Men	26,205	220,155	2,499	11.35	Ref.	Ref.	
Women	31,215	270,229	4,804	17.78	1.55 (1.48–1.63)	1.29 (1.22–1.35)	<0.0001
Age
Under 65	14,406	139,775	672	4.81	Ref.	Ref.	
Between 65 and 80	37,607	318,706	5,616	17.62	3.87 (3.57–4.19)	2.61 (2.41–2.83)	<0.0001
Over 80	5,407	31,903	1,015	31.82	8.36 (7.58–9.22)	5.71 (5.16–6.31)	<0.0001
Insurance type
Health insurance	52,408	451,995	6,484	14.35	Ref.	Ref.	
Medical aid	5,012	38,389	819	21.33	1.55 (1.45–1.67)	1.06 (0.98–1.14)	0.1288
Comorbid diseases
Atherosclerosis	3,801	30,269	933	30.82	2.28 (2.13–2.44)	1.14 (1.06–1.22)	0.0003
Atrial fibrillation	5,533	42,740	1,022	23.91	1.78 (1.66–1.90)	1.08 (1.01–1.16)	0.0243
Bipolar disorder	3,695	25,224	2,160	85.63	9.02 (8.57–9.49)	1.21 (1.13–1.30)	<0.0001
Cerebrovascular disease	11,919	86,778	3,625	41.77	4.97 (4.75–5.21)	2.16 (2.05–2.27)	<0.0001
Depression	8,247	58,952	3,637	61.69	8.03 (7.67–8.41)	2.54 (2.40–2.69)	<0.0001
Diabetes mellitus	16,932	135,354	3,302	24.40	2.23 (2.13–2.34)	1.40 (1.33–1.47)	<0.0001
Dyslipidemia	27,533	237,258	4,798	20.22	2.03 (1.93–2.13)	1.46 (1.38–1.56)	<0.0001
Hypertension	34,998	289,386	5,526	19.10	2.21 (2.10–2.33)	1.34 (1.27–1.42)	<0.0001
Parkinson’s disease	1,675	12,199	829	67.96	5.48 (5.10–5.89)	1.31 (1.21–1.42)	<0.0001
Schizophrenia	1,752	10,923	1,119	102.44	9.57 (8.97–10.21)	1.18 (1.10–1.27)	<0.0001
Sleep disorder	6,705	46,658	2,284	48.95	4.77 (4.54–5.01)	1.48 (1.40–1.57)	<0.0001
Traumatic brain injury	1,791	12,303	753	61.20	4.95 (4.59–5.34)	1.31 (1.21–1.42)	<0.0001
Vascular dementia	10,013	76,196	4,254	55.83	8.15 (7.78–8.54)	3.17 (3.01–3.34)	<0.0001
Concurrent medications
Antidepressants	2,533	17,400	1,132	65.06	5.46 (5.13–5.82)	1.21 (1.13–1.30)	<0.0001
Antiepileptics	405	2,710	189	69.74	5.27 (4.56–6.09)	1.36 (1.17–1.57)	<0.0001
Antihistamines	2,251	13,229	979	74.00	6.54 (6.11–6.992)	2.19 (2.04–2.36)	<0.0001
Antiparkinsonian agents	570	3,601	381	105.80	8.60 (7.75–9.53)	1.28 (1.14–1.43)	<0.0001
Antipsychotics	3,691	23,943	2,353	98.28	12.50 (11.90–13.13)	2.12 (1.97–2.28)	<0.0001
Antispasmodics	589	2,561	463	180.79	18.49 (16.81–20.34)	2.69 (2.43–2.97)	<0.0001
Benzodiazepines	4,354	28,465	1,756	61.69	5.75 (5.45–6.07)	1.48 (1.39–1.57)	<0.0001
Beta–blockers	14,498	120,723	2,506	20.76	1.61 (1.54–1.69)	1.13 (1.07–1.18)	<0.0001
Bladder antimuscarinics	2,422	16,746	1,177	70.29	5.96 (5.59–6.34)	1.95 (1.82–2.08)	<0.0001
CCB (Dihydropyridine)	15,813	135,062	2,794	20.69	1.62 (1.55–1.70)	1.09 (1.04–1.15)	0.0009
CCB (Non–dihydropyridine)	5,128	43,905	828	18.86	1.30 (1.21–1.39)	1.10 (1.03–1.19)	0.0091
HMG–CoA reductase inhibitors	25,227	226,480	3,922	17.32	1.31 (1.25–1.37)	0.83 (0.79–0.88)	<0.0001
Skeletal muscle relaxants	771	4,191	549	130.99	11.68 (10.70–12.75)	1.95 (1.78–2.15)	<0.0001
Zolpidem	2,380	14,746	1,224	83.01	7.55 (7.10–8.03)	1.32 (1.23–1.42)	<0.0001

CCB, calcium channel blocker; CI, confidence interval; HMG-CoA, 3-hydroxy-3-methylglutaryl coenzyme A; HR, hazard ratio; N, number; RAS, renin-angiotensin system. ^a^Incident Alzheimer’s disease per 1,000 person-years. ^b^Adjusted for sex, age, type of insurance, comorbid disease, concurrent medications, and follow-up duration.

**Figure 2:**
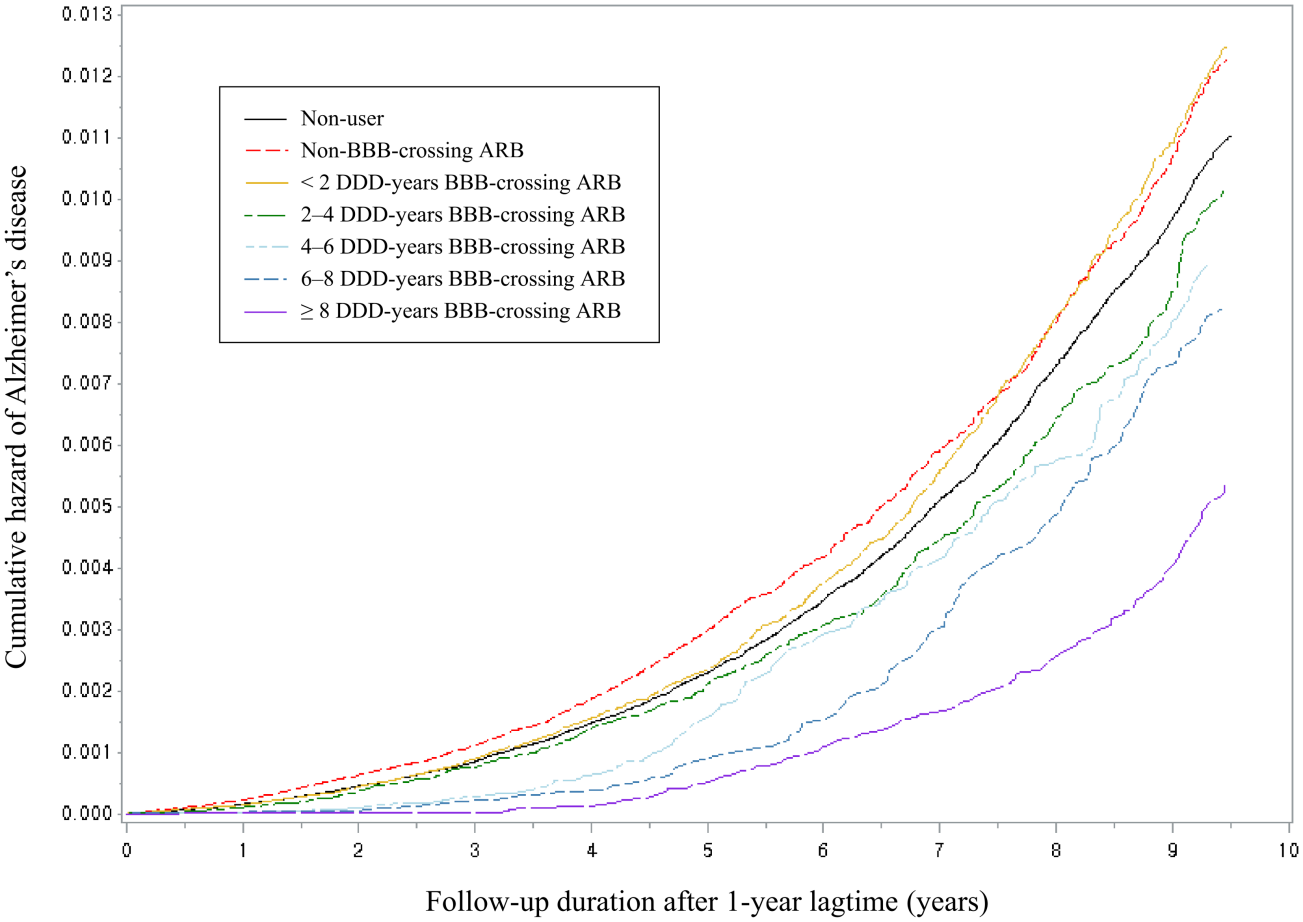
Kaplan–Meier curves for the cumulative hazard of Alzheimer’s disease with a 2 defined daily dose (DDD)-year interval by blood–brain barrier (BBB)-crossing angiotensin II receptor blockers (ARBs).

**Figure 3:**
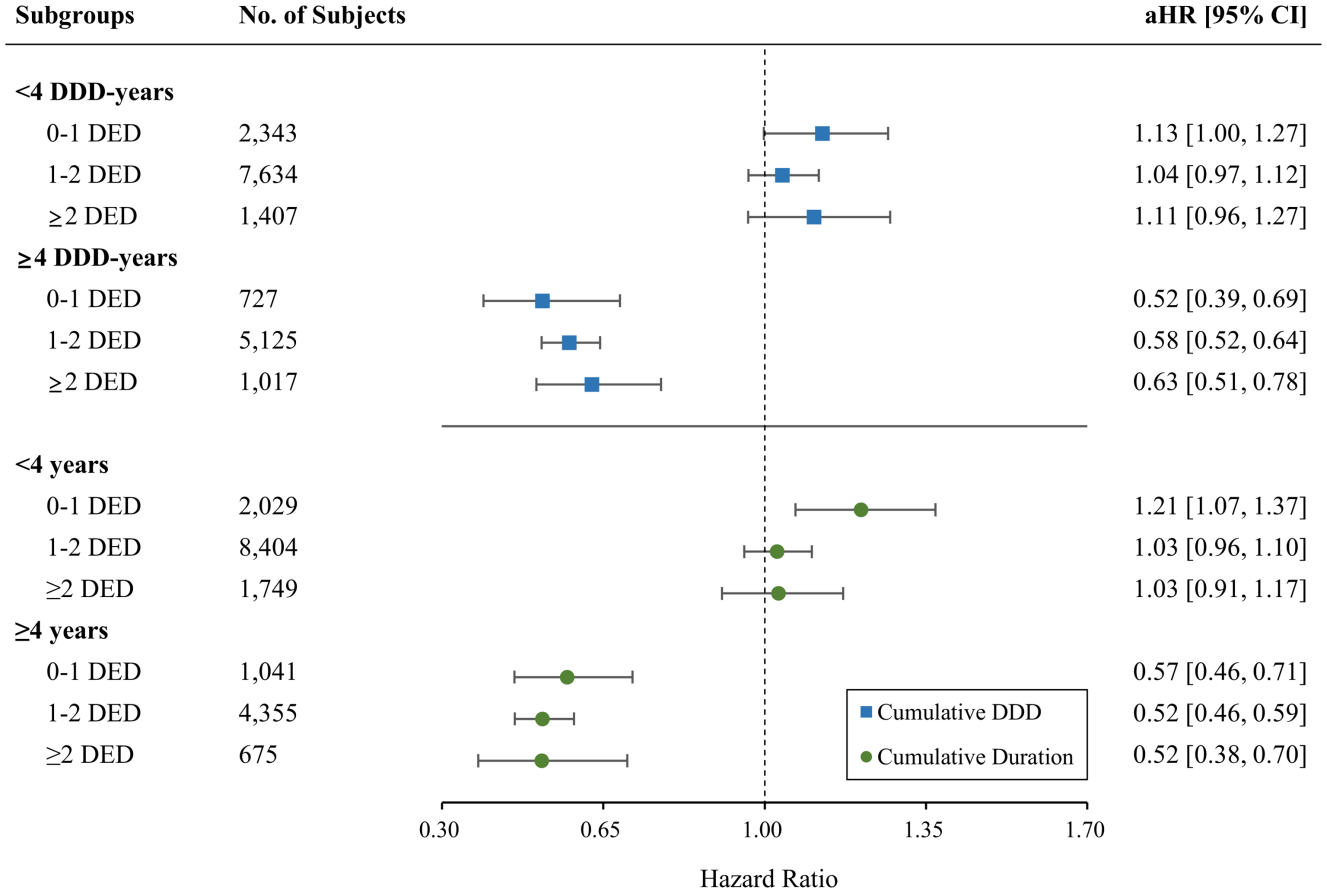
Comparison of the risk of incident Alzheimer’s disease for blood–brain barrier (BBB)-crossing angiotensin II receptor blockers (ARBs) according to the daily equivalent dose (DED) by cumulative daily defined dose (DDD) and duration. aHR, adjusted hazard ratio; CI, confidence interval.

A significant reduction in the risk of AD was observed with ARB use (aHR = 0.94; 95% CI = 0.90–0.99). ACEIs did not show significant association with the AD incidence (aHR = 1.03; 95% CI = 0.97–1.10). Regarding the risk of AD based on the type of RAS inhibitor and BBB permeability, only the use of BBB-crossing ARBs demonstrated a significant protective effect (aHR = 0.83; 95% CI = 0.78–0.88; Table 3). BBB-crossing ARBs were subdivided for additional analyzes based on cumulative DDD, cumulative exposure duration, and daily equivalent dose. Responses depending on cumulative DDD, and duration were observed at 1-year intervals (Supplementary Table S3). Longer exposure to BBB-crossing ARBs was significantly associated with a gradual reduction in AD risk in the trend analysis (*p* < 0.001). The Kaplan–Meier curves of the cumulative hazard according to the cumulative DDD of BBB-crossing ARBs with a 2-year interval are shown in Figure 2. Both ≥4 years of cumulative DDD and ≥ 4 years of cumulative exposure duration showed significantly reduced AD incidence, regardless of daily equivalent dose (Figure 3). The subgroup analysis showed that ARBs were superior to ACEIs in AD risk both in men and women, and there was no difference in the protective effect of BBB-crossing ARBs between men and women (Supplementary Table S4).

**Table 3:** Risk of Alzheimer’s disease by renin-angiotensin system inhibitor type and blood–brain barrier permeability (*N* = 57,420).

	Number of subjects^a^	Person-years	Number of events	Incidence rate^b^	Adjusted HRs (95% CI)^c^
RAS classification					
ACEI	10,933	90,602	1,350	14.90	1.03 (0.97–1.10)
ARB	26,336	227,659	3,269	14.36	0.94 (0.90–0.99)
RAS classification & BBB permeability					
Poor BBB-crossing ACEI	2,690	21,941	368	16.77	1.18 (1.06–1.31)
BBB-crossing ACEI	9,122	75,801	1,094	14.43	1.03 (0.96–1.10)
Poor BBB-crossing ARB	21,252	185,191	2,634	14.22	0.98 (0.93–1.04)
BBB-crossing ARB	18,253	161,827	2,097	12.96	0.83 (0.78–0.88)

ACEI, angiotensin-converting enzyme inhibitor; ARB, angiotensin II receptor blocker; BBB, blood–brain barrier; CI, confidence interval; HR, hazard ratio; RAS, renin-angiotensin system. ^a^The number of subjects in each category of drug type is not mutually exclusive, as the definition of each number of subjects was based on the population who used that drug at least once. ^b^Incident Alzheimer’s disease per 1,000 person-years. ^c^Adjusted for sex, age, type of insurance, comorbid disease, concurrent medications, and follow-up duration.

### Sensitivity analyzes

Sensitivity analyzes for the AD risk of BBB-crossing ARB use for ≥4 DDD years are presented in Table 4. In all sensitivity analyzes with index date shift, lag time extension, outcome definition change, and exclusion criteria expansion, the aHRs of incident AD in users of BBB-crossing ARBs ≥4 DDD-years remained significantly lower than those in RAS inhibitor non-users. Sensitivity analyzes for AD risk of BBB-crossing ARB use <4 DDD-years are shown in Supplementary Table S5.

**Table 4:** Sensitivity analyzes for the risk of Alzheimer’s disease in ≥4 DDD-years of BBB-crossing ARB users.

	Number of subjects	Person-years	Number of events	Incidence rate^a^	Adjusted HRs (95% CI)^b^
Index date shift					
July 1, 2009 (main)	6,869	67,460	507	7.52	0.59 (0.53–0.65)
July 1, 2010	2,387	21,451	174	8.11	0.62 (0.53–0.73)
July 1, 2011	1,729	13,977	121	8.66	0.68 (0.56–0.83)
Lag time extension					
1-year lagged (main)	6,869	67,460	507	7.52	0.59 (0.53–0.65)
3-year lagged	6,347	62,795	435	6.93	0.64 (0.57–0.71)
5-year lagged	5,516	55,517	316	5.69	0.73 (0.64–0.83)
Outcome definition switch					
ICD-10 + neuropsychiatry subject code + ≥2 drug prescriptions (main)	6,869	67,460	507	7.52	0.59 (0.53–0.65)
ICD-10 + ≥2 drug prescriptions	6,853	66,973	663	9.90	0.59 (0.55–0.65)
ICD-10 + neuropsychiatry subject code	6,830	66,802	655	9.81	0.59 (0.54–0.64)
Exclusion criteria expansion					
Main	6,869	67,460	507	7.52	0.59 (0.53–0.65)
Main + presence of PD diagnostic code	6,607	64,984	454	6.99	0.59 (0.53–0.65)
Main + concurrent use of ACEI and ARB	6,459	63,550	479	7.54	0.61 (0.55–0.67)

ACEI, angiotensin-converting enzyme inhibitor; ARB, angiotensin II receptor blocker; BBB, blood–brain barrier; CI, confidence interval; DDD, defined daily dose; HR, hazard ratio; ICD, International Classification of Diseases; PD, Parkinson’s disease. ^a^Incident Alzheimer’s disease per 1,000 person-years. ^b^Adjusted for sex, age, type of insurance, comorbid disease, concurrent medications, follow-up duration, and ACEI.

## Discussion

As the protective effect of antihypertensive agents on cognitive decline beyond their blood pressure-lowering effects has emerged, the potential effect of reducing the risk of AD *via* the renin-angiotensin system has been demonstrated in animal and human studies (Li et al., 2010; Davies et al., 2011; Barthold et al., 2018; Abiodun and Ola, 2020). However, the neuroprotective effects of RAS inhibitors reported in previous studies have been conflicting (Ohrui et al., 2004; Sink et al., 2009; Hebert et al., 2013; Hsu et al., 2013; O’Caoimh et al., 2014; Qiu et al., 2014; Wharton et al., 2015). In this nationwide population-based cohort study, patients with IHD who used BBB-crossing ARBs had a lower risk of incident AD than those who did not use RAS inhibitors. Notably, our study showed a significant reduction in the risk of incident AD in patients who used BBB-permeable ARBs at higher cumulative doses. While previous studies have focused on comparing the effects of ARBs and ACEIs (Marcum et al., 2022) or BBB permeability within RAS inhibitors (Hebert et al., 2013; Qiu et al., 2014; Ho et al., 2021), to the best of our knowledge, this is the first study to simultaneously assess risk reduction considering both BBB permeability and cumulative doses. Our results were robust owing to a valid study design with a long-term follow-up based on nationwide study samples with appropriate comparisons and sensitivity analyzes.

In this study, we revealed that the use of ARBs, but not ACEIs, was associated with a reduced risk of AD. This result is consistent with some clinical studies that have shown the advantageous effect of ARBs over ACEIs in reducing AD risk (Li et al., 2010; Davies et al., 2011; Barthold et al., 2018; Marcum et al., 2022). Additionally, a number of animal studies have supported this difference in protective effect by suggesting potential underlying mechanisms. ACEIs target the angiotensin-converting enzyme (ACE), which is responsible for converting Ang I to Ang II, thus attenuating AT1R and AT2R activation, whereas ARBs selectively block the Ang II/AT1R axis (Gebre et al., 2018). AT1R activation induces oxidative stress, neuroinflammation, and apoptosis, whereas AT2R counteracts AT1R-mediated neurodegeneration by various mechanisms (Lanz et al., 2010; Faraco et al., 2016; Abiodun and Ola, 2020). Hence, the blockade of AT1R by ARBs may induce indirect activation of the Ang II/AT2R axis to provide neuroprotection (Mogi and Horiuchi, 2013). Enhancement of cognitive function by direct stimulation of AT2R was also demonstrated in the animal study (Jing et al., 2012). Moreover, the benefits of ARBs can be attributed to the conversion of Ang II into Ang IV and Ang (1–7), which are selective for AT4R and MASR, respectively. AT4R has been suggested to have a positive effect on cerebral blood flow, memory, and neuroprotection (Näveri et al., 1994; Royea and Hamel, 2020), whereas enhancement of the Ang (1–7)/MASR axis has been reported to have a potential anti-inflammatory effect and facilitate hippocampal long-term potentiation (Hellner et al., 2005; Wright and Harding, 2019). Several studies have also found that the expression of ACE and ACE2 is related to a decreased amyloid-beta (Aβ) load (Hemming and Selkoe, 2005; Zou et al., 2007; Kehoe et al., 2016). However, further translational investigation is essential to confirm the association between the neuroprotective effects of ARBs and Aβ pathology (Loera-Valencia et al., 2021).

Our analyzes indicated that the use of BBB-crossing ARBs was associated with a reduced risk of AD. The finding was especially significant as CI did not overlap with those of the other three types of RAS inhibitors. Additional benefit of using BBB-crossing RAS inhibitors has been demonstrated in previous studies assessing the effect on cognitive function and MCI to AD conversion (Wharton et al., 2015; Ouk et al., 2021). Another longitudinal study, which investigated the effect of ACEIs depending on central exposure, reported no significant association between BBB-crossing ACEIs and AD and a risk of incident AD with poor BBB-crossing ACEIs (Sink et al., 2009). However, a previous meta-analysis (Ho et al., 2021) assessing the effect of BBB-crossing RAS inhibitors on seven cognitive domains reported that poor BBB-crossing RAS inhibitors demonstrated a better effect in the attention domain compared to that of BBB-crossing RAS inhibitors. As previous studies on cognitive decline and incident AD by BBB permeability of RAS inhibitors largely focused on ACEIs (Ohrui et al., 2004; Sink et al., 2009; Hebert et al., 2013; O’Caoimh et al., 2014; Ouk et al., 2021), our results on BBB-crossing ARBs are noteworthy, but the benefit of using BBB-crossing ARBs as potential drugs for preventing AD should be carefully interpreted. In addition to BBB-crossing effects, some *in vitro*/vivo and animal studies have reported that some BBB-crossing ARBs, such as telmisartan, showed partial peroxisome proliferator-activated receptors (PPAR) gamma activation effects that have beneficial effects on cognitive functions (Mogi et al., 2008; Pang et al., 2012; Garg et al., 2021). However, the PPAR-gamma activation effect of ARBs is still controversial, with limited evidence for the attenuation of cognitive functions in only some ARBs (Benson et al., 2004; Erbe et al., 2006; Kajiya et al., 2011). Therefore, further comprehensive studies that have considered the PPAR-gamma binding affinities on ARBs, as well as the BBB-crossing characteristics, are needed.

Remarkably, the significantly reduced risk of AD by BBB-crossing ARBs was robust in patients with a larger cumulative dose or longer duration, regardless of the daily equivalent dose. These results implied that the cumulative exposure duration was a more crucial factor in the neuroprotective effect of ARBs than the daily exposure dose. Risk-reducing effect of ARBs on AD with larger cumulative dose and longer exposure were also demonstrated in a previous longitudinal study, supporting our findings (Chiu et al., 2014). Considering that antihypertensive drugs are generally used for an extended period and our results showed a cumulative effect of BBB-crossing ARBs on AD, they could be suggested as promising targets for drug repurposing. The current treatment for AD shows modest effects only on symptoms (Atri, 2019; Cummings et al., 2020), and the efficacy of the newly approved drug, aducanumab, is also controversial (Whitehouse et al., 2022). Moreover, midlife hypertension has been associated with an increased risk of AD, and blood pressure control is a modifiable risk factor for cognitive decline (Lennon et al., 2019). Taken together, BBB-crossing ARBs might be a promising disease-modifying drug option for reducing the risk of AD in patients with cardiovascular diseases, such as hypertension.

In the subgroup analysis, no difference in the protective effect of BBB-crossing ARBs was identified between men and women. A study by Barthold et al. reported that ARBs were superior to ACEIs in risk of AD incidence for white men and women, but no association was observed for the black and Hispanic populations (Barthold et al., 2018). Estrogen lowers AT1R expression, prevents the production and action of angiotensin II, and decreases NADPH-oxidase activity and expression of neuroinflammatory markers (De Silva and Faraci, 2012; O’Hagan et al., 2012; Rodriguez-Perez et al., 2015). Aging men with aromatization of androgens to estrogens have a higher estrogen level than that of aging women with dramatic ovarian loss of 17β-estradiol (Rosario et al., 2011). A study on the pathophysiology of sex differences in the protective effect of ARB owing to race is needed. Moreover, further studies in other Asian countries are needed to confirm the sex difference in the protective effect of ARBs in Asians.

This study has several limitations. First, this study used a secondary claims database; therefore, we could not verify detailed clinical information, including symptoms, body weight, blood pressure, smoking, alcohol intake, education level, and genetic factors, such as APOE ε4. In addition, the limitation related to the accuracy of incident AD needs to be considered because the outcome variable was identified based on ICD-10 diagnostic codes. Given that the diagnosis of AD is based on the patient’s symptoms (Atri, 2019) and the protective effects of RAS inhibitors have been reported to vary according to cognitive symptoms (Ho et al., 2021), our results should be carefully interpreted. However, we attempted to use the medication prescription claims along with the diagnostic codes for enhanced accuracy of outcome definitions. Moreover, we adopted various outcome definitions in sensitivity analyzes to confirm the robustness of the study results. Second, the possibility of a selection bias cannot be neglected, as our study population was selected based on a very short identification period of 6 months. Moreover, we could not consider active comparators and make a direct comparison of the effect of drugs with different mechanisms, such as beta-blockers, CCBs, or thiazides, because antihypertensive agents are usually used in combination. To minimize this selection bias, we balanced RAS inhibitor users and non-users by PS matching and adjusted for various confounders using rigorous definitions. Moreover, our sensitivity analysis by shifting the index date provided comparable results. Third, it is difficult to generalize the study results to the entire population, as our study population included patients with IHD, who have a high cardiovascular profile. RAS inhibitors, possessing strong vascular effects, has been used for treating various cardiovascular diseases, and conflicting results have been reported on the neuroprotective effect of ARBs, depending on the study population. Further research on the effects of RAS inhibitors on AD in patients with various cardiovascular diseases is required. Finally, the duration or cumulative doses of concomitant medications could not be considered in our study. Instead of considering the variability of confounder status by using time-varying Cox regression, this study used the precise definition of comorbidities and concurrent medications that appeared at least once every year during the follow-up period.

To the best of our knowledge, this is the first longitudinal study to demonstrate the effect of BBB-crossing ARBs on the incidence of AD with cumulative dose and duration subgroups using a population-based cohort. In this study, we highlighted the neuroprotective effect of ARBs, particularly BBB-crossing ARBs, on AD. Additionally, we present a novel finding of the protective effects against AD conferred by long-term use of BBB-crossing ARBs. In addition to existing evidence, these results are expected to provide valuable insights for AD-targeted drug development.

## Data availability statement

The datasets presented in this article are not readily available because the primary data analyzed in this study are handled and stored by the Health Insurance Review and Assessment Service. Requests to access the datasets should be directed to Health Insurance Review and Assessment Service, https://www.hira.or.kr.

## Ethics statement

The studies involving human participants were reviewed and approved by Institutional Review Board (IRB) of Yonsei University. Written informed consent for participation was not required for this study in accordance with the national legislation and the institutional requirements.

## Author contributions

HWL and SK contributed for study design, data analysis, data interpretation, and writing of the manuscript. YMY contributed for study conceptualization, data interpretation, critical revision of the manuscript, and supervision of the study. YJ and YK contributed to data analysis and manuscript revision. BSY contributed to clinical interpretation of the data and critical revision of the manuscript. All authors contributed to the article and approved the submitted version.

## Funding

This work was supported by the National Research Foundation of Korea (NRF) grant funded by the Ministry of Science and ICT (MSIT) of the Korea government (No. 2020R1G1A110120513).

## Conflict of interest

The authors declare that the research was conducted in the absence of any commercial or financial relationships that could be construed as a potential conflict of interest.

## Publisher’s note

All claims expressed in this article are solely those of the authors and do not necessarily represent those of their affiliated organizations, or those of the publisher, the editors and the reviewers. Any product that may be evaluated in this article, or claim that may be made by its manufacturer, is not guaranteed or endorsed by the publisher.

## Supplementary material

The Supplementary material for this article can be found online at: https://www.frontiersin.org/articles/10.3389/fnagi.2023.1137197/full#supplementary-material
