# Supplementary material for: Neuroprotective effect of angiotensin II receptor blockers on the risk of incident Alzheimer’s disease: A nationwide population-based cohort study
Source: Front Aging Neurosci. 2023 Mar 6;15:1137197. doi: 10.3389/fnagi.2023.1137197 (PMC10025478; doi:10.3389/fnagi.2023.1137197)
Supplement: Supplementary file 1 [file Data_Sheet_1.docx]

**1.1 SUPPLEMENTARY FIGURES**

**
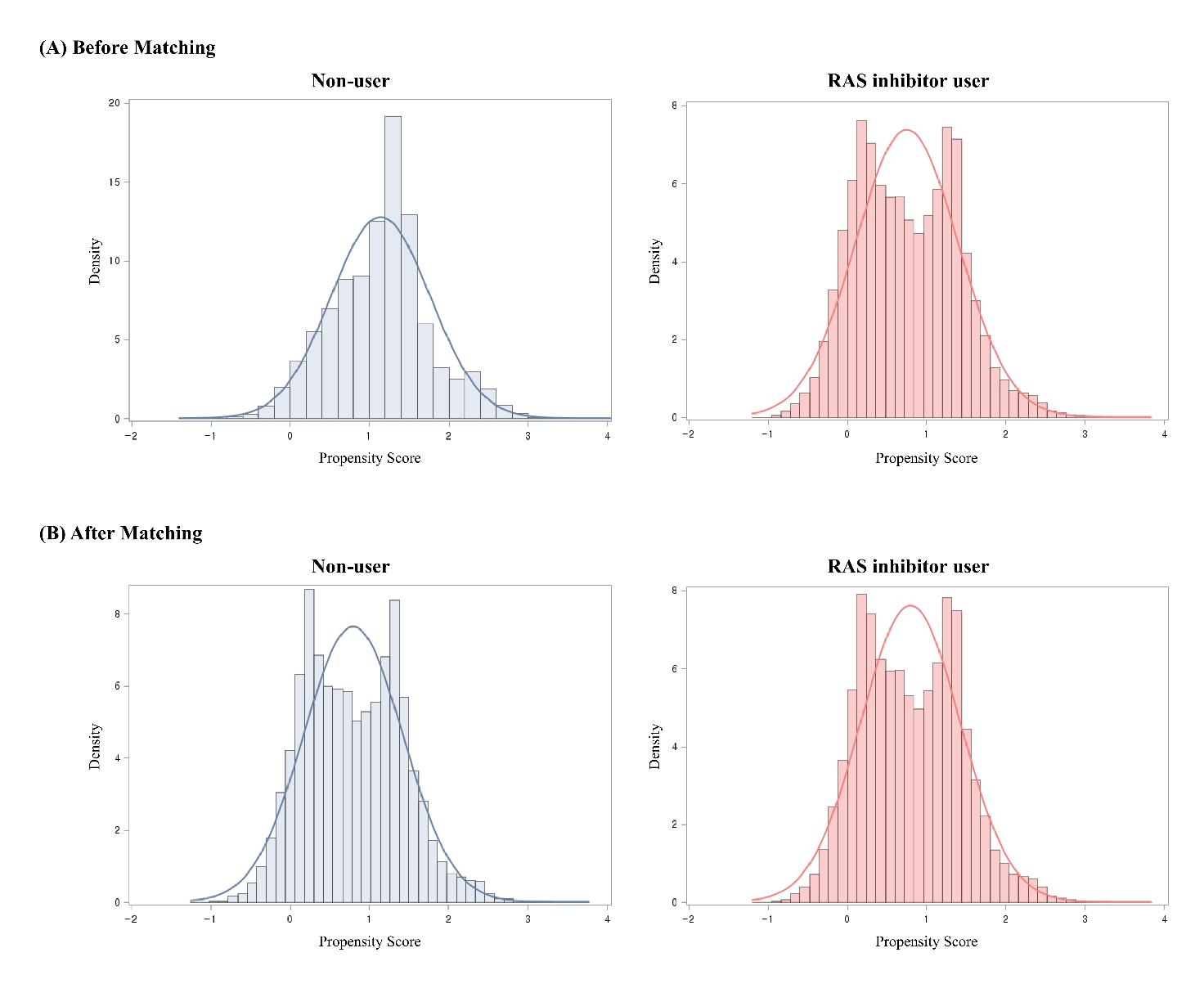
**

**Supplementary Figure S1.** Distribution of propensity score in users and non-users of renin-angiotensin system (RAS) inhibitors before (A) and after (B) the matching.


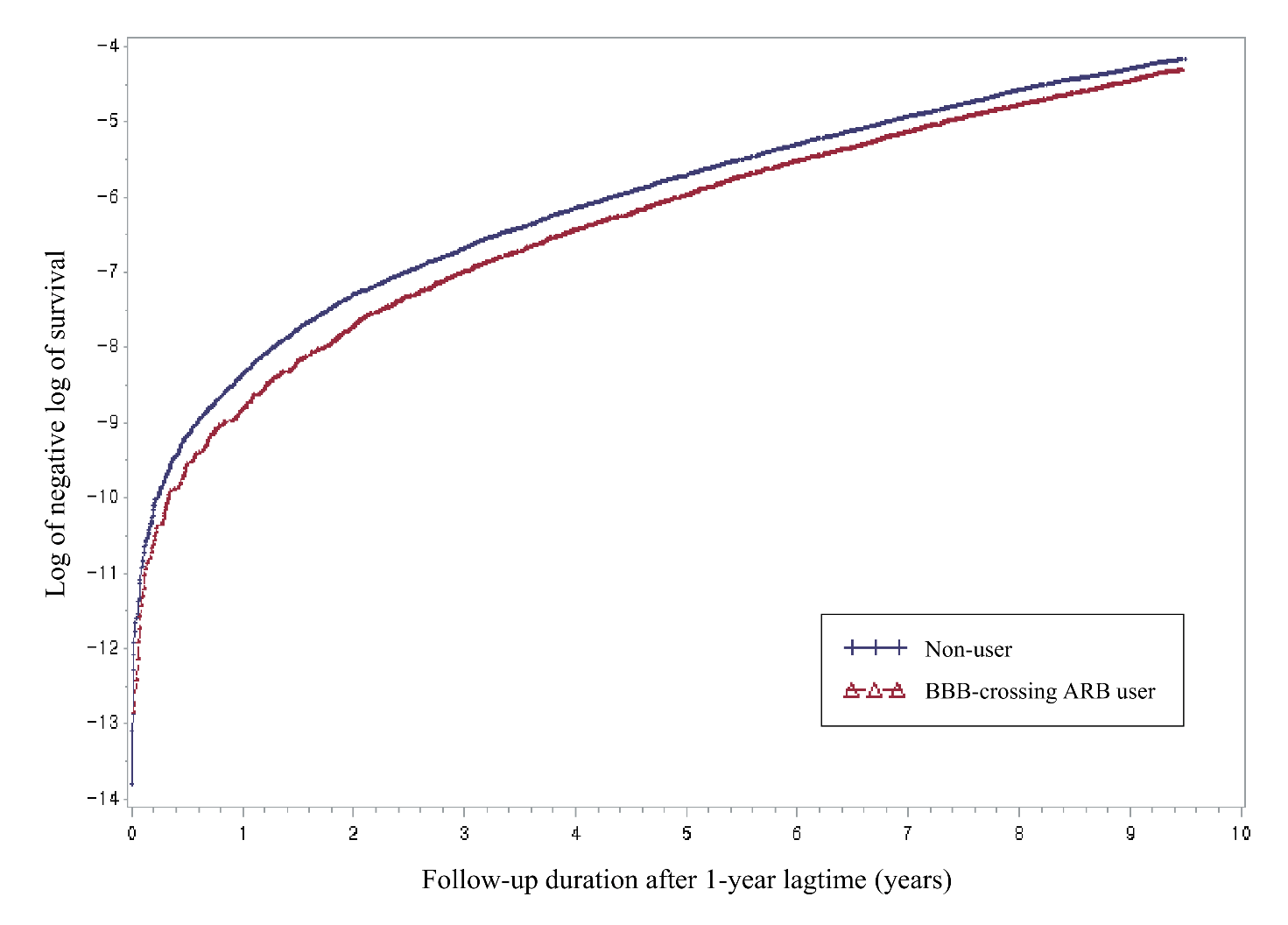


**Supplementary Figure S2.** Log minus log plot of Kaplan-Meier estimation for graphical validation of the proportional hazard assumption in blood-brain barrier (BBB)-crossing angiotensin II receptor blockers (ARBs).

**1.2 SUPPLEMENTARY TABLES**

**Supplementary Table S1.** List of ICD-10 codes for comorbid diseases.

| **Disease** | **ICD-10 codes** |
| --- | --- |
| Atherosclerosis | I70 |
| Atrial fibrillation | I48 |
| Bipolar disorder | F30–F31, F34, F34.0, F34.8, F34.9 |
| Cerebrovascular disease^a^ | I60–I69, G45–G46 |
| Depression | F32–F33, F34.1 |
| Diabetes mellitus | E11 |
| Dyslipidemia | E78 |
| Hypertension | I10 |
| Parkinson’s disease | G20 |
| Schizophrenia | F20–F26, F28–F29 |
| Sleep disorder | G47, F51 |
| Traumatic brain injury | S01.9, S06–S09 |
| Vascular dementia | F01–F03, G31 |

ICD-10, International Classification of Disease 10^th^ Revision.

^a^Composite of hemorrhagic infarction, ischemic cortical infarction, and vasculopathy.

**Supplementary Table S2.** List of drugs for concurrent medications.

| **Drug class** | **Drug name** |
| --- | --- |
| Antidepressants | Amitriptyline, Amoxapine, Clomipramine, Doxepin, Imipramine, Nortriptyline, Paroxetine, Protriptyline, Trimipramine |
| Antiepileptics | Carbamazepine, Oxcarbazepine |
| Antihistamines | Chlorpheniramine, Clemastine, Dimenhydrinate, Diphenhydramine, Doxylamine, Hydroxyzine, Piprinhydrinate, Promethazine, Triprolidine |
| Antiparkinsonian agents | Benztropine, Biperiden, Procyclidine, Trihexyphenidyl |
| Antipsychotics | Chlorpromazine, Chlorprothixene, Clozapine, Levomepromazine, Olanzapine, Perphenazine, Pimozide, Quetiapine, Trifluoperazine |
| Antispasmodics | Alverine, Atropine, Cimetropium, Chlordiazepoxide, Clidinium-Chlordiazepoxide, Dicyclomine, Glycopyrrolate, Hyoscine |
| Benzodiazepines | Alprazolam, Clonazepam, Clorazepate, Diazepam, Estazolam, Flurazepam, Lorazepam, Triazolam, |
| Beta-blockers | Amosulalol, Arotinolol, Atenolol, Betaxolol, Bevantolol, Bisoprolol, Carteolol, Carvedilol, Celiprolol, Esmolol, Labetalol, Metoprolol, Nadolol, Nebivolol, Propranolol, Sotalol |
| Bladder antimuscarinics | Fesoterodine, Flavoxate, Oxybutynin, Propiverine, Solifenacin, Tolterodine, Trospium |
| CCB (dihydropyridine) | Amlodipine, Barnidipine, Benidipine, Cilnidipine, Efonidipine, Felodipine, Isradipine, Lacidipine, Lercanidipine, Manidipine, Nicardipine, Nifedipine, Nilvadipine, Nimodipine, Nisoldipine, Nitrendipine |
| CCB (non-dihydropyridine) | Diltiazem, Verapamil |
| Skeletal muscle relaxants | Carisoprodol, Chlorzoxazone, Cyclobenzaprine, Methocarbamol, Orphenadrine, Tizanidine |
| HMG-CoA reductase inhibitors | Atorvastatin, Fluvastatin, Lovastatin, Pitavastatin, Pravastatin, Rosuvastatin, Simvastatin |
| Zolpidem | Zolpidem |

CCB, calcium channel blocker; HMG-CoA, 3-hydroxy-3-methylglutaryl coenzyme A.

**Supplementary Table S3.** Risk of Alzheimer’s disease by cumulative DDD and duration of BBB-crossing ARB use.

|  | **Number of subjects** | **Person-years** | **Number of events** | **Incidence rate^a^** | **Adjusted HRs (95% CI)^b^** |
| --- | --- | --- | --- | --- | --- |
| **RAS inhibitor non-users** | 28,710 | 244,738 | 3,689 | 15.07 | Ref. |
| **Cumulative DDD** |  |  |  |  |  |
| 0-1 DDD-years | 6,471 | 52,478 | 944 | 17.99 | 1.12 (1.04–1.21) |
| 1-2 DDD-years | 2,048 | 16,814 | 284 | 16.89 | 1.13 (1.00–1.29) |
| 2-3 DDD-years | 1,576 | 13,480 | 208 | 15.43 | 0.96 (0.84–1.11) |
| 3-4 DDD-years | 1,289 | 11,597 | 154 | 13.28 | 0.89 (0.75–1.05) |
| 4-5 DDD-years | 1,038 | 9,639 | 100 | 10.37 | 0.73 (0.60–0.90) |
| 5-6 DDD-years | 954 | 9,010 | 93 | 10.32 | 0.75 (0.61–0.93) |
| 6-7 DDD-years | 826 | 7,937 | 74 | 9.32 | 0.64 (0.51–0.81) |
| 7-8 DDD-years | 685 | 6,765 | 62 | 9.16 | 0.67 (0.52–0.87) |
| 8-9 DDD-years | 547 | 5,440 | 39 | 7.17 | 0.47 (0.35–0.65) |
| 9-10 DDD-years | 548 | 5,459 | 25 | 4.58 | 0.40 (0.27–0.60) |
| ≥ 10 DDD-years | 2,271 | 23,211 | 114 | 4.91 | 0.45 (0.37–0.54) |
| *P for trend* |  |  |  |  | <0.001 |
| **Cumulative exposure duration** |  |  |  |  |  |
| 0-1 years | 6,688 | 54,173 | 974 | 17.98 | 1.09 (1.01–1.18) |
| 1-2 years | 2,232 | 18,192 | 328 | 18.03 | 1.24 (1.11–1.40) |
| 2-3 years | 1,806 | 15,557 | 216 | 13.88 | 0.87 (0.75–0.99) |
| 3-4 years | 1,456 | 13,259 | 174 | 13.12 | 0.93 (0.80–1.09) |
| 4-5 years | 1,230 | 11,631 | 112 | 9.63 | 0.69 (0.57–0.84) |
| 5-6 years | 1,003 | 9,742 | 89 | 9.14 | 0.68 (0.55–0.84) |
| 6-7 years | 911 | 9,058 | 76 | 8.39 | 0.59 (0.47–0.75) |
| 7-8 years | 754 | 7,651 | 59 | 7.71 | 0.64 (0.49–0.82) |
| 8-9 years | 593 | 6,096 | 37 | 6.07 | 0.48 (0.35–0.66) |
| 9-10 years | 676 | 7,024 | 28 | 3.99 | 0.32 (0.22–0.47) |
| ≥ 10 years | 904 | 9,445 | 4 | 0.42 | 0.05 (0.02–0.13) |
| *P for trend* |  |  |  |  | <0.001 |
| **Daily equivalent dosage** |  |  |  |  |  |
| 0-1 DDD/day | 3,070 | 26,945 | 363 | 13.60 | 0.97 (0.87–1.09) |
| 1-2 DDD/day | 12,759 | 114,198 | 1,429 | 12.77 | 0.86 (0.81–0.92) |
| ≥ 2 DDD/day | 2,424 | 20,685 | 305 | 14.83 | 0.91 (0.81–1.02) |

ACEI, angiotensin-converting enzyme inhibitor; ARB, angiotensin II receptor blocker; BBB, blood-brain barrier; CI, confidence interval; DDD, defined daily dose; HR, hazard ratio; RAS, renin-angiotensin system.

^a^Incident Alzheimer’s disease per 1,000 person-years.

^b^Adjusted for sex, age, type of insurance, comorbid disease, concurrent medications, follow-up duration, and ACEI.

**Supplementary Table S4.** Subgroup analysis for the risk of Alzheimer’s disease by sex.

|  | **Male (N=26,205)** | | **Female (N=31,215)** | |
| --- | --- | --- | --- | --- |
|  | **Adjusted HRs (95% CI)^a^** | **p-value** | **Adjusted HRs (95% CI)^a^** | **p-value** |
| **RAS classification** | | | | |
| ACEI | 0.99 (0.89–1.10) | 0.8957 | 1.06 (0.98–1.15) | 0.1327 |
| ARB | 0.94 (0.87–1.03) | 0.1839 | 0.94 (0.89–1.00) | 0.0547 |
| **RAS classification & BBB permeability** | | | | |
| Poor BBB-crossing ACEI | 1.19 (0.99–1.43) | 0.0715 | 1.18 (1.04–1.35) | 0.0131 |
| BBB-crossing ACEI | 1.01 (0.91–1.13) | 0.8010 | 1.05 (0.97–1.15) | 0.2318 |
| Poor BBB-crossing ARB | 1.02 (0.92–1.13) | 0.6830 | 0.96 (0.89–1.03) | 0.2234 |
| BBB-crossing ARB | 0.79 (0.71–0.88) | <0.0001 | 0.85 (0.79–0.92) | <0.0001 |
| **Cumulative DDD of BBB-crossing ARB** | | | | |
| <4 DDD-years | 1.05 (0.94–1.18)^b^ | 0.4119 | 1.08 (0.99–1.17)**^b^** | 0.0700 |
| ≥4 DDD-years | 0.58 (0.48–0.69)**^b^** | <0.0001 | 0.59 (0.53–0.66)^b^ | <0.0001 |

ACEI, angiotensin-converting enzyme inhibitor; ARB, angiotensin II receptor blocker; BBB, blood-brain barrier; CI, confidence interval; DDD, defined daily dose; HR, hazard ratio.

^a^ Adjusted for age, type of insurance, comorbid disease, concurrent medications, and follow-up duration.

^b^ACEI only users were excluded (male n=24,851, female n=30,195) and adjusted for age, type of insurance, comorbid disease, concurrent medications, follow-up duration, and ACEI.

**Supplementary Table S5. S**ensitivity analyses for the risk of Alzheimer’s disease in <4 DDD-years of BBB-crossing ARB use.

|  | **Number of subjects** | **Person-years** | **Number of events** | **Incidence rate^a^** | **Adjusted HRs (95% CI)^b^** |
| --- | --- | --- | --- | --- | --- |
| **Index date shift** | | | | | |
| July 1, 2009 (main) | 11,384 | 94,368 | 1,590 | 16.85 | 1.07 (1.01–1.15) |
| July 1, 2010 | 3,663 | 28,592 | 503 | 17.59 | 1.06 (0.95–1.19) |
| July 1, 2011 | 2,611 | 18,649 | 352 | 18.88 | 1.23 (1.07–1.40) |
| **Lag time extension** | | | | | |
| 1-year lagged (main) | 11,384 | 94,368 | 1,590 | 16.85 | 1.07 (1.01–1.15) |
| 3-year lagged | 9,163 | 83,206 | 1,105 | 13.28 | 1.07 (0.98–1.15) |
| 5-year lagged | 7,110 | 69,234 | 647 | 9.35 | 1.06 (0.95–1.17) |
| **Outcome definition switch** | | | | | |
| ICD-10 + neuropsychiatry subject code + ≥2 drug prescriptions (main) | 11,384 | 94,368 | 1,590 | 16.85 | 1.07 (1.01–1.15) |
| ICD-10 + ≥2 drug prescriptions | 11,397 | 92,960 | 2,121 | 22.82 | 1.11 (1.05–1.17) |
| ICD-10 + neuropsychiatry subject code | 11,403 | 93,566 | 1,931 | 20.64 | 1.08 (1.02–1.15) |
| **Exclusion criteria expansion** | | | | | |
| Main | 11,384 | 94,368 | 1,590 | 16.85 | 1.07 (1.01–1.15) |
| Main + presence of PD diagnostic code | 10,797 | 89,702 | 1,373 | 15.31 | 1.07 (1.00–1.15) |
| Main + concurrent use of ACEI and ARB | 10,444 | 87,474 | 1,453 | 16.61 | 1.08 (1.01–1.16) |

ACEI, angiotensin-converting enzyme inhibitor; ARB, angiotensin II receptor blocker; BBB, blood-brain barrier; CI, confidence interval; DDD, defined daily dose; HR, hazard ratio; ICD, International Classification of Diseases; PD, Parkinson’s disease.

^a^Incident Alzheimer’s disease per 1,000 person-years.

^b^Adjusted for sex, age, type of insurance, comorbid disease, concurrent medications, follow-up duration, and ACEI.
